# Micro/Nano Structural Investigation and Characterization of Mussel Shell Waste in Thailand as a Feasible Bioresource of CaO

**DOI:** 10.3390/ma16020805

**Published:** 2023-01-13

**Authors:** Wiranchana Srichanachaichok, Dakrong Pissuwan

**Affiliations:** 1 Materials Science and Engineering Graduate Program, Faculty of Science, Mahidol University, Bangkok 10400, Thailand; nw.wiranchana@gmail.com; 2 Nanobiotechnology and Nanobiomaterials Research Laboratory, School of Materials Science and Innovation, Faculty of Science, Mahidol University, Bangkok 10400, Thailand; 3 Materials Science and Nano Engineering Undergraduate Program, Faculty of Science, Mahidol University, Bangkok 10400, Thailand

**Keywords:** calcium carbonate (CaCO_3_), calcium oxide (CaO), green mussel shells, mixed shell powder, shell waste

## Abstract

Mussel shell waste, which is regularly disposed by households, restaurants, markets, or farms, causes environmental problems worldwide, including in Thailand, because of its long decomposing time. Owing to a large amount of calcium (Ca) content from calcium carbonate (CaCO_3_) in mussel shell waste, many Thai local businesses grind the shell waste into powder and sell it as a source of Ca. Generally, these powdered waste shells are a mixture of various types of mussel shell waste. In this study, we investigated and characterized powdered mixed waste shells sold in a local Thai market (called mixed shell powder) and ground shells from waste green mussel shells (called green mussel shells) prepared in the laboratory after calcination at different temperatures (800 °C, 900 °C, and 1000 °C). Mixed shell powder containing five different types of mussel shells and green mussel shells were calcined for 2 h and 3 h, respectively. The time used for calcination of mixed shell powder and green mussel shells was different due to the different particle sizes of both shell wastes. We found that an optimal temperature of 1000 °C completely converted CaCO_3_ to CaO in both samples. The nanoscale size of CaO was detected at the surface of calcined shells. These shell wastes can be used as a bioresource of CaO.

## 1. Introduction

Molluscan aquaculture is a worldwide sustainable food resource that has increased global molluscan food production. Abundant shells are generated after food production, which impacts the environment [1]. Methods to transform molluscan shells into valuable products have been investigated to reduce the adverse environmental impact. As mentioned previously, a major component of molluscan shells is calcium carbonate (CaCO_3_). It has been reported that biominerals in molluscan shells contain approximately 95% CaCO_3_, 5% proteins, and polysaccharides [2]. CaCO_3_ can be transformed into lime (calcium oxide, CaO) after an optimal heat treatment process called calcination [3,4]. The calcination reaction is shown in Equation (1) [5,6].

CaCO_3_ → CaO + CO_2_ (1)

Owing to the presence of CaO, calcined molluscan shells can be used in many applications, such as in the construction industry and agriculture. Moreover, many studies revealed that CaO can be used as a bioactive material in food packaging [7] and has a high potential to kill bacteria [8,9] and fungi [10].

In Thailand, molluscan shells have been consumed for over 100 years, resulting in an increase in molluscan farming and production [11]. Consequently, there has been an increase in molluscan shell waste. It was reported that around 50,000 tons of shell waste from the seafood industry are produced per year [12]. This shell waste can pollute the area where the waste is discarded because of a bad smell from decomposing of residual attached meat on the molluscan shell [13]. Furthermore, some discarded shells are dumped in landfill or in the sea. Without control of the dumped discarded shells, ecosystems of soil, water, and sea can increase the risk of damaging ecosystems [14]. This issue motivated us to search for a method to utilize discarded molluscan shells. The transformation of discarded shells into beneficial materials will reduce the volume and increase the value of shell waste. It is necessary to investigate the components of shell waste for proper utilization. In this study, we characterized the two forms of discarded shells: (i) discarded green mussel shells because Thailand is a major producer [11], and (ii) mixed shell powder sold in local Thai markets. Due to the high amount of shell waste in Thailand, local people grind the waste shells into powder without applying heat and supply the shell powder as feed or fertilizers to farmers. This has become a conventional technique to utilize shell waste in Thailand. However, if the mixed shell powder can be transformed to have more value, this resource will provide more benefits to people in communities. Hence, we investigated biominerals (CaCO_3_ and CaO) in discarded green mussel shells and mixed shell powder. The mixed shell powder mainly contains clams, chin mussels, cockles, and scallops. We also investigated the effect of calcination temperatures on discarded green mussel shells and mixed shell powder. In addition, the transformation of the calcium phase, nanostructure of calcined shells, and the characteristics of discarded green mussel shells and mixed shell powder before and after calcination were studied.

## 2. Materials and Methods

### 2.1. The Preparation of Shell Waste

**Figure 1:**
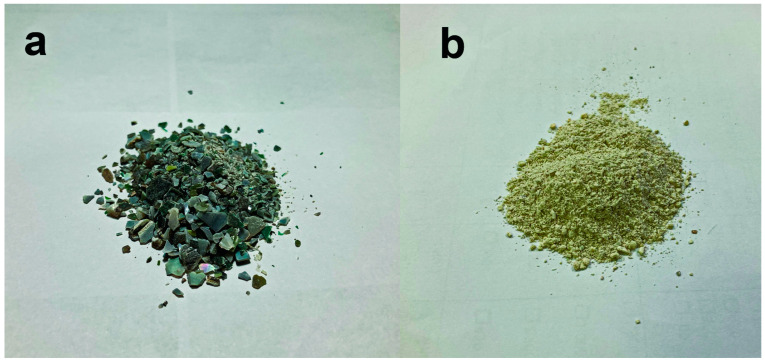
Prepared mussel shell waste. (**a**) Crushed green mussel shells and (**b**) mixed shell powder from the local market.

Two types of shell waste were used in the present study: (i) crushed green mussel shells prepared from discarded shells collected from a fresh market (Figure 1a), and (ii) mixed shell powder (the mixture of discarded clams, chin mussels, cockles, and scallops) procured from a local market seller in the Samut Songkhram Province (Figure 1b), Thailand. The green mussel shells were washed to remove dust and impurities and sterilized using an autoclave. After autoclaving, green mussel shells were dried in an oven at 100 °C and crushed into powder using a stone mortar for further investigation. In the case of the mixed shell powder, it was procured from the local market as powder and used directly.

### 2.2. The Calcination of Molluscan Waste Shells

Crushed green mussel shells and mixed shell powder were calcined at 800 °C, 900 °C, and 1000 °C in a furnace for 3 h and 2 h, respectively. The calcination time was 2 h for mixed shell powder because of the smaller size of the shell particles than that of the crushed green mussel shell particles. After calcination, samples were cooled to room temperature. Both types of shell waste were ground to a fine powder using an agate mortar. The calcined waste shells were dried and later stored in a zip-lock bag at room temperature to avoid interaction with moisture.

### 2.3. The Characterization of Calcined and Non-Calcined Waste Shells

The thermal decomposition patterns of both waste shells were investigated by thermogravimetric analysis (TGA). The samples were prepared by weighing 20 mg of each of the crushed green mussel shells and mixed shell powder. These samples were placed in the pan of a thermogravimetric analyzer. Continuous heating from room temperature to 1000 °C at a heating rate of 10 °C min^−1^ was programmed during the analysis.

### 2.4. X-ray Diffraction (XRD) and Fourier Transform Infrared (FTIR) Spectroscopy Analysis

XRD was employed to investigate the crystal structure of the calcined and non-calcined waste shells. Finely ground shells were placed on the holder and analyzed by an X-ray diffractometer using CuK_α_ radiation over a 2θ range from 25° to 70°. FTIR spectroscopy was used to investigate the functional groups of CaO formed after calcination. The shell samples were prepared using the same process as that used for XRD analysis. FTIR analysis was recorded between 500 cm^−1^ and 4000 cm^−1^.

### 2.5. Scanning Electron Microscopy (SEM)

The morphologies of crushed green mussel shells and mixed shell powder before and after calcination and then grinding by an agate motar were investigated by SEM (SU8000, Hitashi, Tokyo, Japan). Next, each powder was placed on the conductive carbon adhesive tape attached to a stub. The sample was sputter coated with platinum-palladium using a sputter coater before imaging. The SEM images were taken at 10.0 kV with a secondary electron detector.

### 2.6. Elemental and Statisical Analysis

Elements in both waste shells (before and after calcination) were analyzed using X-ray fluorescence (XRF). Finely powdered green mussel shells and mixed shell powder, 12.5 g each, were placed on an XRF sample cup covered with a transparent film at the bottom of the cup to investigate the components of the waste shells. The mean value of measured elements and the standard error of the mean was calculated from three replicates.

## 3. Results and Discussion

### 3.1. The Decomposition Characteristics of Green Mussel Shells and Mixed Shell Powder

**Figure 2:**
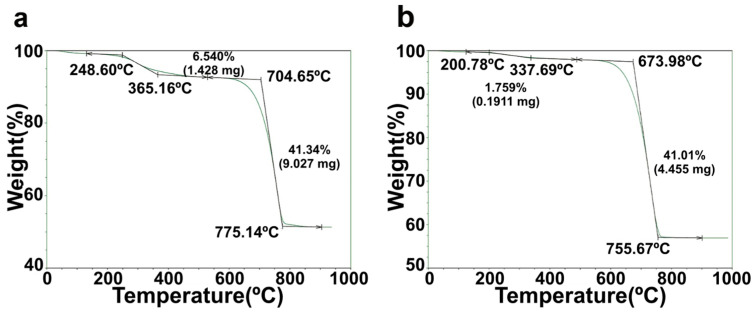
The TGA patterns of (**a**) crushed green mussel shells and (**b**) mixed shell powder.

The TGA patterns of green mussel shells and mixed shell powder are shown in Figure 2. The green mussel shell sample was decomposed in two steps. The initial step with a small weight loss of ~6.5% of green mussel shells was detected in the temperature range of 249–365 °C, which can be due to the removal of the moisture content [6,15,16]. A major weight loss of green mussel shells was detected in the following step from approximately 705 °C and ending at ~775 °C. At this step, it exhibits a rapid weight loss of ~41.3% (Figure 2a). These results are similar to those of a previous study reporting that the decomposition of volatile minerals occurs with the release of carbon dioxide (CO_2_) within this temperature range [16]. A small weight loss of ~1.8% was detected in the temperature range of 201–338 °C for mixed shell powder, while a rapid weight loss of ~41.4% occurred in the temperature range of 674–756 °C (Figure 2b). The weight loss in the second step was much higher than that in the first stage for both samples. An increase in temperature in the second step can be attributed to the decomposition of volatile minerals in both waste shell samples. The transformation of CaCO_3_ to CaO occurs in the second stage. The weights of both samples were almost constant at the final stage at temperatures ≥775 °C for green mussel shells and ≥756 °C for mixed shell powder. The weights of both waste shell samples remained stable at 900 °C. This indicates that total decomposition reaches an equilibrium state at this temperature; CaCO_3_ completely decomposed to CaO and ash was obtained as a residue. A comparison between green mussel shells and mixed shell powder reveals that the latter requires a lower temperature to transform the crystalline phases of shells. This can be due to the difference in sizes of crushed green mussel shells and mixed shell powder [16]. Small-sized particles have a larger surface area than large-sized particles. Therefore, this could be the reason why the temperature during the first two stages of mixed shell powder was lower than that of crushed green mussel shells.

The fraction of decomposed green mussel shells and mixed shell powder (α) was calculated using Equation (2) [17]. The weight loss with respect to the total weight loss of the samples was used to calculate the fraction of decomposed green mussel shells and mixed shell powder. The initial weight is denoted by Wi and Wt denotes the current weight. The final weight is denoted as Wf.

(2) $$\alpha =\frac{\mathrm{Wi}-\mathrm{Wt}}{\mathrm{Wi}-\mathrm{Wf}}$$

**Figure 3:**
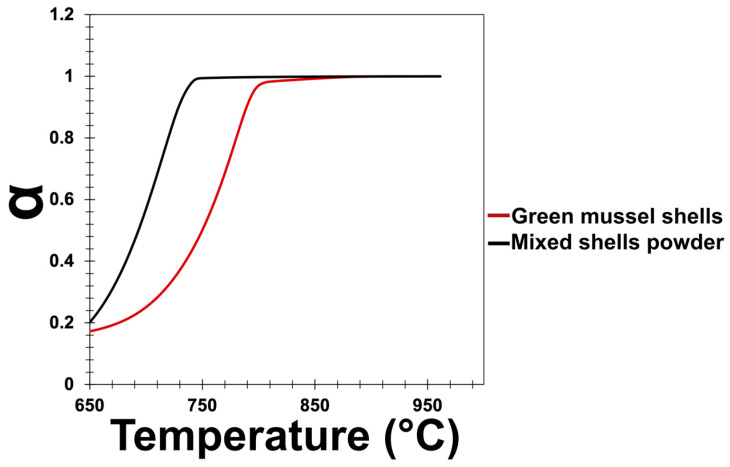
The fraction of decomposed green mussel shells and mixed shell powder.

From the graph of the fraction of decomposed green mussel shells and mixed shell powder (Figure 3), it is clear that the large-sized green mussel shell particles require a higher temperature to completely decompose than the small-sized mixed shell powder particles.

### 3.2. The Crystalline Phases of Green Mussel Shells and Mixed Shell Powder during Calcination

**Figure 4:**
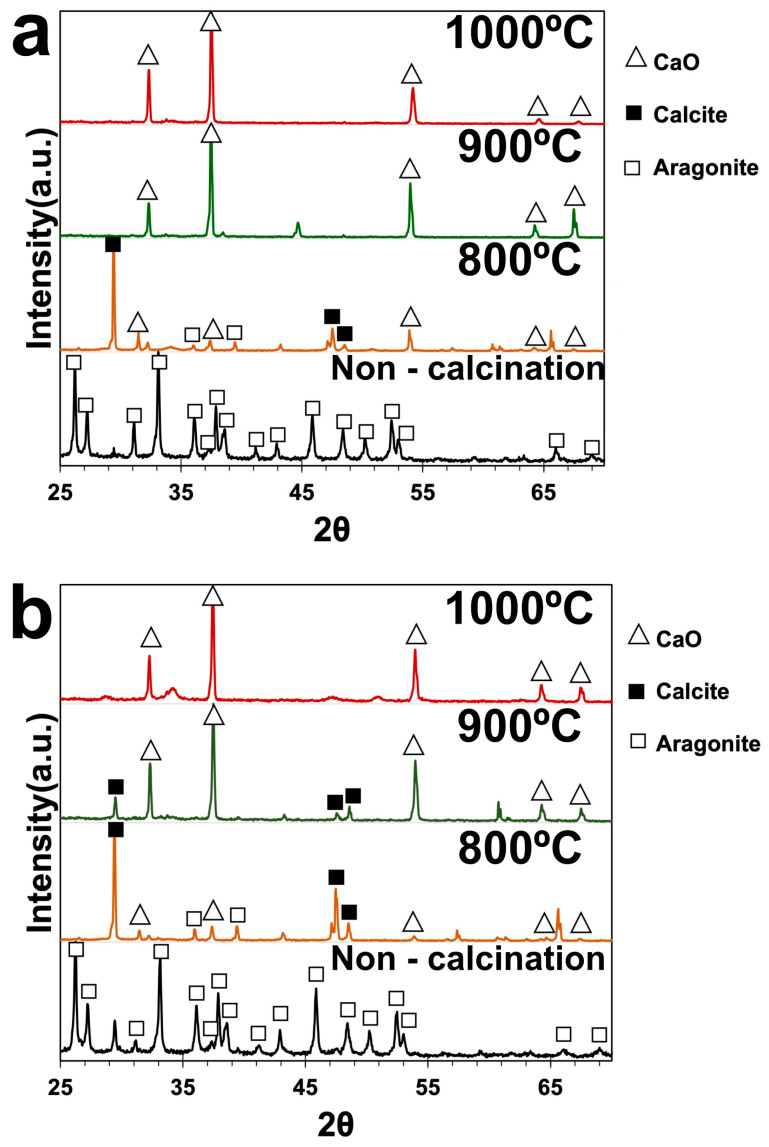
The XRD spectrum of (**a**) green mussel shells and (**b**) mixed shell powder.

XRD analysis was used to determine the phases of green mussel shells and mixed shell powder. Figure 4 shows the XRD patterns of green mussel shells without calcination with strong diffraction peaks corresponding to the aragonite phase of CaCO_3_ at 2ϴ values of 26°, 27°, 31°, 33°, 36°, 37.5°, 38.5°, 41°, 43°, 45.5°, 48°, 50°, 52°, 53°, 66°, and 69°. These peaks are in agreement with the standard JCPDS File No. 01-071-2396 [18,19] (Figure 4a). A similar diffraction pattern was observed for non-calcined mixed shell powder (Figure 4b). This indicates that non-calcined green mussel shells and mixed shell powder have a similar aragonite phase of CaCO_3_. The calcined green mussel shells and mixed shell powder at 900 °C and 1000 °C reveal characteristic diffraction peaks of CaO (cubic phase of CaO) at 2ϴ values of 32°, 37°, 54°, 64°, and 67° [19]. However, the diffraction peaks of calcined green mussel shells and mixed shell powder at 800 °C indicate the aragonite phase of CaCO_3_ and CaO. The presence of characteristic peaks at 2ϴ values of 29.5°, 47°, and 48° indicates a calcite phase of CaCO_3_, as previously reported [20]. This shows that the calcite phase is also detected in green mussel shells and mixed shell powder after calcination at 800 °C. It was reported that the transformation of aragonite to calcite occurs before the thermal decomposition of CaCO_3_ after heat induction [21]. The characteristic peaks of the calcite phase of CaCO_3_ continue to appear after the calcination of mixed shell powder at 900 °C for 2 h (Figure 4b). However, the characteristic peak of CaCO_3_ is absent in green mussel shells at this temperature. This can be due to the different contents and structures of shell types. Muhdarina et al. [22] reported that a hard shell layer can affect the decomposition of CaCO_3_ to CaO during calcination. In our study, we found that the complete decomposition of CaCO_3_ to CaO occurs at 1000 °C for 3 h in green mussel shells and for 2 h in mixed shell powder.

### 3.3. FTIR Spectra

**Figure 5:**
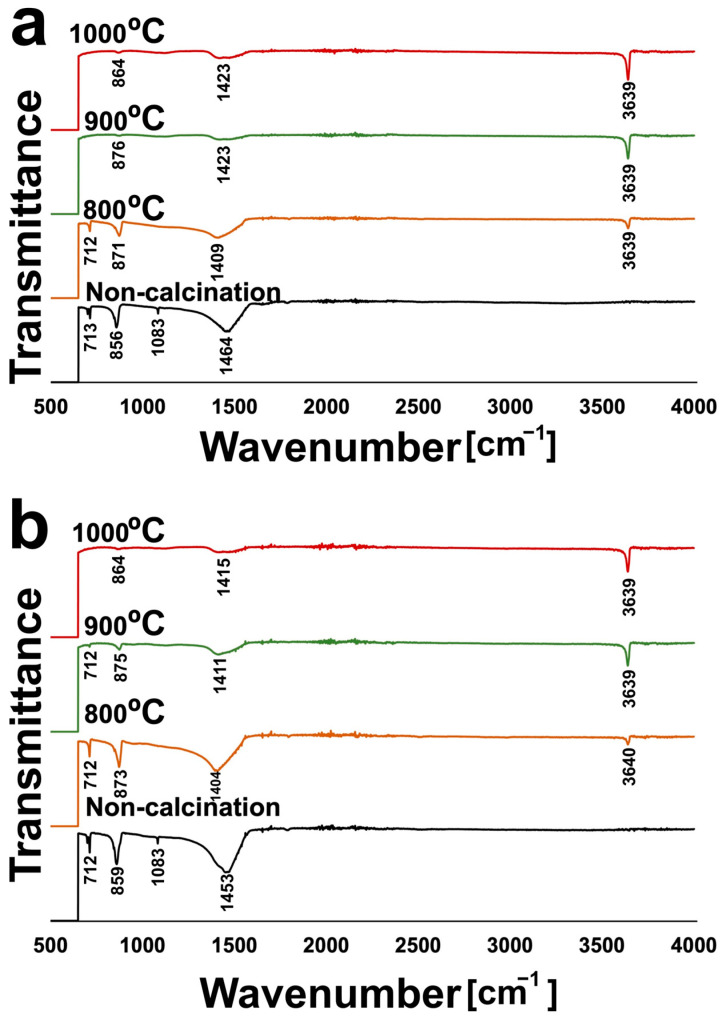
The FTIR spectra of (**a**) green mussel shells (3 h calcination) and (**b**) mixed shell powder (2 h calcination).

As shown in Figure 5, similar FTIR spectra of non-calcined green mussel shells and mixed shell powder were recorded in the range of 500–4000 cm^−1^. The major absorption bands of non-calcined green mussel shells were detected at 712 cm^−1^ and 856 cm^−1^. These bands are similar to those detected for non-calcined mixed shell powder (712 cm^−1^ and 859 cm^−1^). The other sharp major peaks appeared at 1463 cm^−1^ and 1453 cm^−1^ of non-calcined green mussel shells and mixed shell powder, respectively, demonstrating the presence of CaCO_3_ [23]. The broadening of spectrum peaks was observed on calcined green mussel shells and mixed shell powder at different temperatures (Figure 5a,b), indicating the conversion of CaCO_3_ into CaO [24]. All the samples calcined at 800–1000 °C show a sharp band at 3639 cm^−1^. This band is involved in the OH stretching vibration mode of water absorbed on the surface of CaO [22,25]. Figure 5 exhibits that peak heights at ~3639 cm^−1^ increase with the increasing temperature. This implies a high-temperature effect on the decomposition of CaCO_3_ to CaO.

### 3.4. Element Compositions

The elemental composition of green mussel shells and mixed shell powder with/without calcination was detected by XRF. As shown in Table 1, approximately 97.74% and 98.09% of Ca are present in green mussel shells and mixed shell powder without calcination, respectively. A previous report also demonstrated ~94% of the Ca content found in green mussel shells [26]. An increase in Ca content up to ~99% was detected in green mussel shells (99.33%) and mixed shell powder (99.42%) after calcining at 1000 °C. A slightly lower percentage of Ca (~98%) was detected in calcined green mussel shells and mixed shell powder at 800 °C and 900 °C, respectively, in comparison to both waste shell samples calcined at 1000 °C (~99%). Small amounts of other minerals were also detected in green mussel shells and mixed shell powder with or without calcination (Table 1). The XRF results show that calcination can affect the reduction of sulphur (S) in green mussel shells. As shown in Table 1, 0.29% of S detected in non-calcined green mussel shells decreased to 0.09% in calcined green mussel shells. This indicates the impact of heat on the reduction in S [27]. However, mixed shell powder samples calcined at all temperatures have the similar amount of S at 0.08–0.09%. The small weight change of S in non-calcined mixed shell powder (0.1%) is still unclear. However, the size of particles might be involved in this. Small reductions of Fe, Mn, and Sr are present in both calcined samples. The K and Sn contents of green mussel shells and mixed shell powder significantly decreased after calcination at 1000 °C. Ca is the major element detected in green mussel shells and mixed shell powder samples. The results are similar to the previously reported work that the major element in bivalves and mussel shells is Ca [28,29].

### 3.5. The Morphology of Green Mussel Shells and Mixed Shell Powder

**Figure 6:**
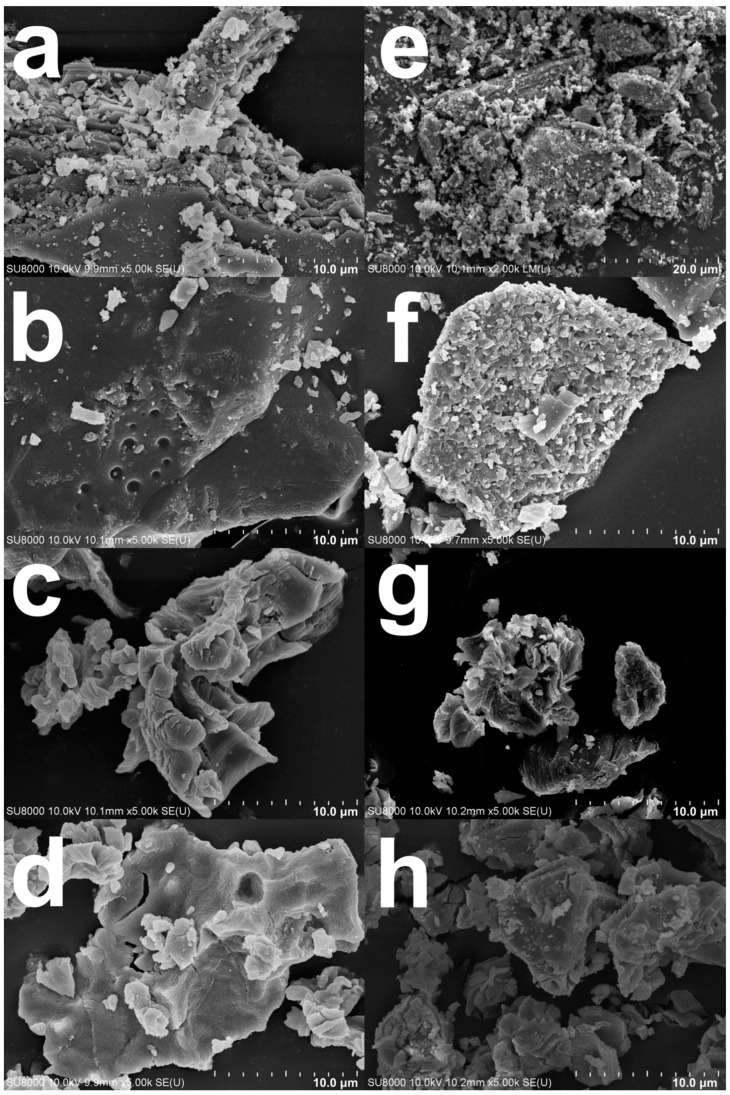
The SEM images of (**a**) green mussel shells without calcination; calcined green mussel shells for 3 h at (**b**) 800 °C, (**c**) 900 °C, and (**d**) 1000 °C; (**e**) mixed shell powder without calcination; and calcinated mixed shell powder for 2 h at (**f**) 800 °C, (**g**) 900 °C, and (**h**) 1000 °C.

The surface morphologies of green mussel shells and mixed shell powder before and after calcination were investigated by using SEM, which reveals similar morphologies. As seen in Figure 6a,e, the SEM images show typical layers and bulky structures of both waste shells without calcination. This kind of structure was also reported in a previous study [23]. Furthermore, irregular and small branching rod shapes were observed in both waste shell samples. These various shapes indicate the presence of aragonite crystals naturally found in CaCO_3_ [30]. In fact, there are various morphologies of aragonite crystals: rod-like, needle-like, dendrite-like, pseudo-hexagonal, and multilayered crystals [31,32]. The surfaces of calcined green mussel shells appear as relief lines (Figure 6b–d) similar to a previous report [6]. Porous structures are shown in calcined green mussel shells (Figure 6b–d) and mixed shell powder (Figure 6f–h). As reported by Nasir and Nazri [23], the calcination can affect the porosity of shell powder.

**Figure 7:**
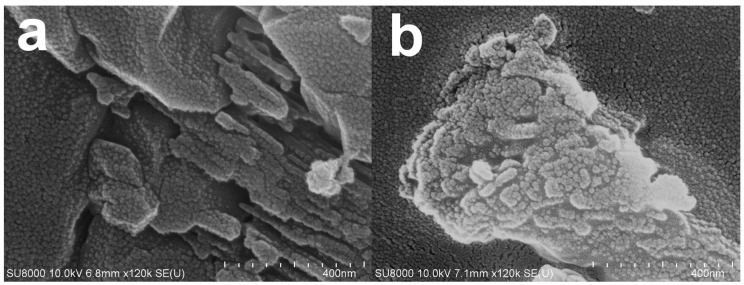
The SEM images of (**a**) green mussel waste shells calcined at 1000 °C for 3 h and (**b**) mixed shell powder calcined at 1000 °C for 2 h.

According to XRD spectra (Figure 4), CaO alone was detected in green mussel shells and mixed shell powder calcined at 1000 °C for 3 h and 2 h, respectively. Therefore, we investigated the CaO structure of both waste shell samples using SEM after calcining at 1000 °C. The sizes of various particles are at the nanoscale level are demonstrated in Figure 7. Similar structures of CaO nanoparticles from natural seashell waste, *Lottioidea reticularis*, after calcination at 900 °C for 4 h were reported by Anand et al. [19]. The appearance of CaO agglomeration of round particles in the range of nanoscales located at the surface was also detected in different types of mussel waste shells, reported by Laonapakul et al. [33]. The comparison between green mussel shells and mixed shell powder after calcination at 1000 °C shows that more regular particles are present in the mixed shell powder (Figure 7b) than those in green mussel shells (Figure 7a), although mixed shell powder was calcined for a shorter time (2 h). This could be due to the difference in the sizes of crushed green mussel shells and mixed waste shell powder.

## 4. Conclusions

Our study demonstrated that the main component of green mussel shells and mixed shell powder is CaCO_3_. The particle size of waste shells has some impact on their structure after calcination. The calcination of crushed green mussel shells and mixed shell powder can convert CaCO_3_ to nanoscale CaO, which can be used as a bioactive compound in many applications. Therefore, green mussel shells and mixed shell powder sold in the local market can be a potential CaO bioresource. Instead of using mixed shell powder as an ingredient for animal feed or fertilizer, an increase in its value as a bioactive material can be achieved through calcination. In addition, the grinding process of shell waste before calcination can reduce the calcination time required to obtain CaO. As shown in our study, at the same calcination temperature, the mixed shell powder required a shorter calcination time than crushed green mussel shells. This study provides new perspectives and directions for increasing the value of shell waste, reducing the amount of shell waste, and adding more value to shell waste as a bioresource of CaO. The CaO obtained from the shell waste can be a valuable source for multiple purposes such as a bioactive antimicrobial agent, catalyst for biodiesel production, and adsorbent.

## Figures and Tables

**Table 1:** The Composition of Elements in Green Mussel Shells and Mixed Shell Powder.

Green Mussel Shells	Composition (wt%)						
	Ca	Fe	K	Mn	S	Sn	Sr
Non-calcination	97.74 ± 0.17	0.26 ± 0.01	0.39 ± 0.01	0.10 ± 0.01	0.29 ± 0.01	0.58 ± 0.06	0.64 ± 0.08
800 °C	98.15 ± 0.03	0.12 ± 0.02	0.39 ± 0.04	0.06 ± 0.01	0.09 ± 0.00	0.60 ± 0.04	0.58 ± 0.04
900 °C	98.26 ± 0.02	0.13 ± 0.02	0.38 ± 0.00	0.07 ± 0.00	0.09 ± 0.01	0.67 ± 0.04	0.41 ± 0.02
1000 °C	99.33 ± 0.01	0.09 ± 0.00	0.00 ± 0.00	0.06 ± 0.01	0.09 ± 0.01	0.00 ± 0.00	0.43 ± 0.01
**Mixed Shell Powder**							
Non-calcination	98.09 ± 0.02	0.32 ± 0.00	0.43 ± 0.01	0.11 ± 0.01	0.10 ± 0.02	0.57 ± 0.08	0.38 ± 0.05
800 °C	98.14 ± 0.48	0.22 ± 0.06	0.28 ± 0.14	0.07 ± 0.01	0.08 ± 0.01	0.37 ± 0.24	0.34 ± 0.04
900 °C	98.61 ± 0.32	0.18 ± 0.02	0.24 ± 0.12	0.07 ± 0.00	0.09 ± 0.00	0.42 ± 0.21	0.38 ± 0.03
1000 °C	99.42 ± 0.03	0.17 ± 0.01	0.00 ± 0.00	0.07 ± 0.01	0.01 ± 0.01	0.00 ± 0.00	0.25 ± 0.02

## Data Availability

Not applicable.
