# Correction: Srichanachaichok, W.; Pissuwan, D. Micro/Nano Structural Investigation and Characterization of Mussel Shell Waste in Thailand as a Feasible Bioresource of CaO. *Materials* 2023, *16*, 805

**DOI:** 10.3390/ma19061263

**Published:** 2026-03-23

**Authors:** Wiranchana Srichanachaichok, Dakrong Pissuwan

**Affiliations:** 1 Materials Science and Engineering Graduate Program, Faculty of Science, Mahidol University, Bangkok 10400, Thailand; nw.wiranchana@gmail.com; 2 Nanobiotechnology and Nanobiomaterials Research Laboratory, School of Materials Science and Innovation, Faculty of Science, Mahidol University, Bangkok 10400, Thailand; 3 Materials Science and Nano Engineering Undergraduate Program, Faculty of Science, Mahidol University, Bangkok 10400, Thailand

In the original publication [1], there was a mistake in Figure 4. The corrected Figure 4 appears below. The authors state that the scientific conclusions are unaffected. This correction was approved by the Academic Editor. The original publication has also been updated.

**Figure 4:**
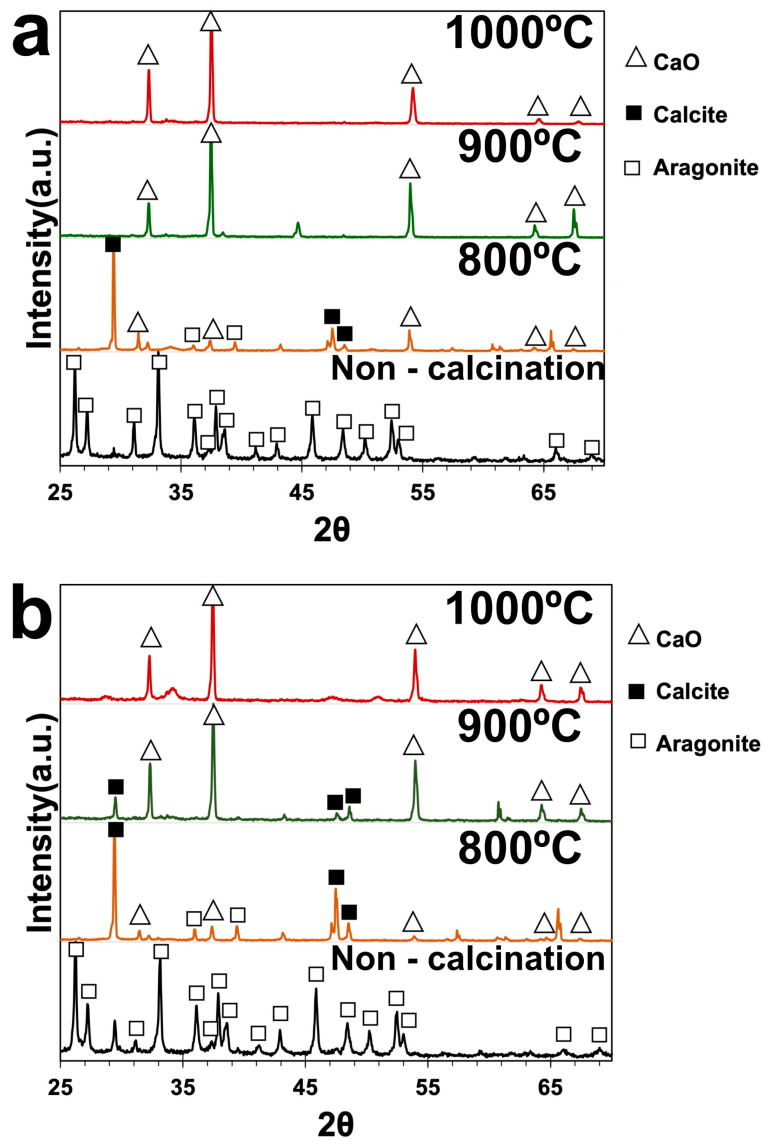
The XRD spectrum of (**a**) green mussel shells and (**b**) mixed shell powder.
